# Study protocol of ‘Prism Adaptation in Rehabilitation’: a randomized controlled trial in stroke patients with neglect

**DOI:** 10.1186/s12883-015-0263-y

**Published:** 2015-02-04

**Authors:** Antonia F Ten Brink, Johanna MA Visser-Meily, Tanja CW Nijboer

**Affiliations:** Brain Center Rudolf Magnus and Center of Excellence for Rehabilitation Medicine, University Medical Center Utrecht and De Hoogstraat Rehabilitation, Rembrandtkade 10, 3583 TM Utrecht, The Netherlands; Department of Experimental Psychology, Helmholtz Institute, Utrecht University, Heidelberglaan 1, 3584 CS Utrecht, The Netherlands

**Keywords:** Unilateral neglect, Prism adaptation, Treatment, Clinical trial, Intervention, Stroke

## Abstract

**Background:**

A frequent disorder after stroke is neglect, resulting in a failure to report or respond to contralesional stimuli. Rehabilitation of neglect is important, given the negative influence on motor recovery, independence in self-care, transfers, and locomotion. Effects of prism adaptation (PA) to alleviate neglect have been reported. However, either small groups or no control group were included and few studies reported outcome measurements on the level of activities of daily living (ADL). The current ongoing RCT investigates the short- and long-term effects of PA in a large population in a realistic clinical setting. Measures range from the level of function to the level of ADL.

**Methods/Design:**

Neglect patients in the sub-acute phase after stroke are randomly assigned to PA (n = 35) or sham adaptation (SA; n = 35). Adaptation is performed for 10 consecutive weekdays. Patients are tested at start of the study, 1 and 2 weeks after starting, and 1, 2, 4 and 12 weeks after ending treatment. Primary objectives are changes in performance on neuropsychological tests and neglect in ADL. Secondary objectives are changes in simulated driving, eye movements, balance, visual scanning and mobility, subjective experience of neglect in ADL and independence during ADL.

**Discussion:**

If effective, PA could be implemented as a treatment for neglect.

**Trial registration:**

This trial is registered at the Dutch Trial Register #NTR3278.

## Background

Unilateral neglect occurs frequently after stroke, resulting in a failure to report or respond to stimulation in contralesional hemispace (25-30% of all stroke patients, [1,2]). In 40% of patients, neglect does not recover after one year and becomes chronic [3]. Functional outcome of stroke patients suffering from neglect is worse than that of stroke patients without neglect [3,4], and motor recovery patterns are slower and more attenuated [5]. As a result, many studies aim at alleviating the symptoms of neglect with different treatments such as visual scan training, limb activation, mental imagery training, sensory stimulation, and prism adaptation. The effectiveness of these treatments remains unproven and more research is needed in a realistic clinical setting [8].

A promising treatment for neglect is PA [6,7,9,10]. PA was first described by Rossetti et al. [11]. Exposure to prisms produces a lateral shift of the visual field so that targets appear displaced. Adaptation to such an optical shift requires a set of successive visuo-motor pointing movements. When the prisms are removed, attention is automatically shifted to the contralesional side. Rossetti et al. [11] demonstrated a significant reduction of spatial neglect following a brief period of PA with rightward prisms. Effects of PA have been reported across clinical tests of neglect, but also in more daily situations, such as wheelchair navigation [12], mental imagery [13], and balance [14]. The beneficial effects of prism adaptation have been reported to last two hours [11,12,15] up to one week [16,17] after a single session, and even up to six weeks following repetitive PA [18-20]. Additionally, long-term prism training has been reported to show long-lasting beneficial effects, from weeks [21-24] up to two years [25] after ending prism adaptation. Notwithstanding these positive results, either small groups or single cases were reported, no control group was included, and/or no measurements at the level of activities of daily living (ADL) were used.

This ongoing study is designed to answer the following primary research question: Can *early* intervention with PA ameliorate neglect both *better* and *earlier* compared to sham adaptation (SA)? Secondary questions are: (1) When are the optimal effects reached?; (2) What is the time course of beneficial effects of an intensive programme of exposure to prisms?; (3) Does PA affects neglect in simulated driving, eye movements, balance, visual scanning and mobility, subjective experience of neglect and independence during ADL?

## Methods

### Design

**Figure 1:**
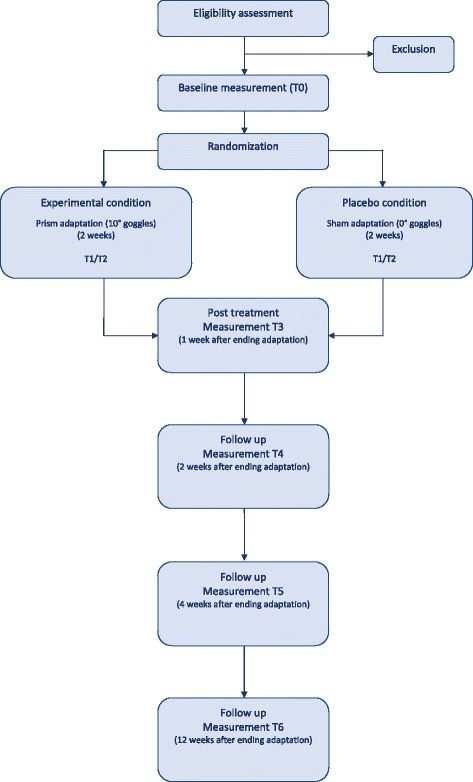
**Procedure.** After baseline measurement (T0), patients are randomized and receive treatment for 10 consecutive weekdays. Patients are tested after 1 week (T1), at end of treatment (T2), and 1, 2, 4 and 12 weeks after treatment (T3-6, respectively).

This RCT compares the effects of PA versus SA, both in addition to usual care (Figure 1). After the baseline measurement, patients will be randomly assigned to one of the two conditions: prism or sham. All patients will receive two weeks of daily treatment (5 days per week). Patients will be tested 7 times in total: at start of the study (T0; baseline), 1 week after starting treatment (T1), 2 weeks after starting treatment/at end of intervention (T2), 1 week after ending treatment (T3), 2 weeks after ending treatment (T4), 4 weeks after ending treatment (T5), and 12 weeks after ending treatment (T6).

This study is conducted according to the principles of the Declaration of Helsinki (59th WMA General Assembly, Seoul, Korea, October 2008) and in accordance with the Medical Research Involving Human Subjects Act (WMO). The study is approved by the ‘Medisch Etische Toetsingscommissie’ of the University Medical Centre Utrecht (#12-183/O).

### Patient population – inclusion and exclusion criteria

We recruit 70 patients, admitted to De Hoogstraat Rehabilitation Centre (the Netherlands). Within the first two weeks of admission, a neuropsychologist administers neglect tests, and a nurse observes neglect in ADL according to the Catherine Bergego Scale (CBS) as standard stroke care. The inclusion criteria of this study are (1) clinical diagnosed symptomatic stroke (ischemic or intracerebral haemorrhagic lesion), first or recurrent; (2) neglect, indicated with neuropsychological neglect tests (Shape Cancellation test or Line Bisection test) and/or CBS; (3) 18–85 years of age; (4) sufficient comprehension and communication; (5) sufficient motivation, and (6) written informed consent. The exclusion criteria are (1) interfering psychiatric disorders and/or substance abuse; (2) expected discharge < 4 weeks; and (3) physically and/or mentally unable to participate. The rehabilitation physician is consulted regarding the exclusion criteria.

### Randomization

Before start of the study, 70 printed cards with the treatment (35 PA and 35 SA) are enveloped. The investigator opens one after the baseline measurement to assign the patient to the stated treatment. Each patient will have an equal chance of being allocated to any of the conditions.

### Treatment

All patients receive the current common rehabilitation programme parallel the treatment.

#### Experimental treatment

The PA procedure is similar to that employed by Rossetti et al. [11], with the exception that it is repeated on 10 consecutive weekdays. Patients wear a pair of goggles fitted with wide-field point-to-point prismatic lenses, inducing a ipsilesional optical shift of 10°. Exposure consists of ±100 fast pointing movements to visual targets presented 10° to the left or right of the body midline at a distance of ±65 cm [26]. A board is held under the chin to prevent viewing of the hand at its starting position, but allowing an unobstructed view of the targets and terminal errors. Next, the after-effect is measured: patients point to the middle target with closed eyes to prevent online adjustment of the pointing movements towards the target due to visual feedback. For successful PA, a contralesional shift of ±3 cm from the target is required. The procedure is repeated when the after-effect is less than 3 cm.

##### Placebo treatment

SA is performed with a pair of goggles with plain lenses (i.e. no optical shift). The procedure is the same as during PA. The ‘after effect’ is tested. No shift is expected.

### Measurements

#### Baseline descriptors

The following admission-to-rehabilitation data are collected: demographics (age, gender, educational level), stroke characteristics (time post-stroke, hemisphere, type, stroke history (first-ever or recurrent), motor function (Motricity Index; MI; [27]), and cognition (Mini-mental state examination; MMSE, [28]).

#### Primary outcomes

Primary endpoints are changes in performance on neuropsychological neglect tests (Star Cancellation, Letter Cancellation, Line Bisection, Landmark Test, Copying, Mental Representation and Symmetrical Photos) and neglect in ADL, as measured with the CBS [29,30]. The CBS is an observation scale for assessment of neglect in 10 everyday activities, and is administered by the physical therapist, occupational therapist and nurse.

#### Secondary outcomes

We administer a simple driving simulation task [31], and compute the average position on the road and the average deviation (swinging). Meanwhile we measure eye movements. To objectify balance, patients are asked to sit and/or stand on a Nintendo Wii™ Balance Board [14]. Visual scanning and mobility is assessed with the Mobility Assessment Course (MAC; [32]), which measures the extent to which patients visually scan targets while walking or wheelchair driving through a corridor. The course consists of targets (12 left and 12 right) and directional indicators. We measure subjective experience of neglect with the CBS self-evaluation. Finally, the nurse fills in the Barthel Index (BI; [33]), to measure independence during ADL.

During all sessions, neuropsychological tests, CBS, simulated driving, eye movements and balance are assessed. During even sessions, the MAC, CBS self-evaluation and BI are assessed additionally.

### Data monitoring board

A data monitoring board takes part in this study.

### Sample size estimates

No reliable information on the expected effect of PA on neuropsychological neglect tests or CBS scores is available. An effect size of 0.7 standard deviation was used to estimate the necessary sample size. To identify a difference with a power of 80% and alpha .05 (2-sided), 35 patients per group (70 patients in total) are required for sufficient statistical power.

### Blinding

The investigator who treats and tests the patients is not blind to the treatment, since she has to put on the goggles. The nurses, physical therapist, and occupational therapist filling in the CBS are unaware of the treatment. Patients cannot be blinded to the treatment, since they have to wear the goggles. However, patients are not explicitly told which treatment they receive.

### Statistical analyses

#### Multivariate analysis

Repeated Measures Analyses are performed for each outcome measure separately, with Session (T0-T6) as within-subject variable and Treatment (PA, SA) as between-subject variable. With respect to timing of optimal effects, sessions T0-T1 and T1-T2 are compared. For longitudinal effects, joinpoint analyses are planned [3].

## Discussion

Visuo-spatial neglect is a prevalent disorder and complicates rehabilitation. Despite PA seems a promising intervention, there is not sufficient evidence whether it ameliorates neglect, which withholds implementation. We aim to answer whether PA ameliorates neglect *better* and *earlier* compared to SA. We investigate the intervention in routine practice, to assure that the intervention works in real life settings. Other strengths of this study are the patient sample (i.e. large sample size including both young and older patients), design (i.e. intensive treatment, placebo control arm and randomized design) and range of outcome measures (i.e. ADL measures and follow up) [34].

A weakness of this study is the non-blinding of the investigator. To reduce potential influence of this on the outcomes, instructions are standardized and tasks are computerized when possible. Furthermore, observations are done by therapists who are blinded for the conditions.

To conclude, in case of positive results, we could implement PA as a treatment for neglect in rehabilitation.

## Abbreviations

ADL: Activities of daily living
BI: Barthel
CBS: Catherine Bergego Scale
MAC: Mobility Assessment Course
MI: Motricity
MMSE: Mini-mental state examination
NWO: Netherlands Organization for Scientific Research
PA: Prism adaptation
SA: Sham adaptation
